# Prevalence of Antibodies to 2009 Pandemic Influenza A (H1N1) Virus in German Adult Population in Pre- and Post-Pandemic Period

**DOI:** 10.1371/journal.pone.0021340

**Published:** 2011-06-20

**Authors:** Sandra Dudareva, Brunhilde Schweiger, Michel Thamm, Michael Höhle, Klaus Stark, Gérard Krause, Silke Buda, Walter Haas

**Affiliations:** 1 European Programme for Intervention Epidemiology Training (EPIET), European Centre for Disease Prevention and Control (ECDC), Stockholm, Sweden; 2 Postgraduate Training for Applied Epidemiology (PAE, German FETP), Robert Koch-Institute, Berlin, Germany; 3 National Reference Centre for Influenza, Department of Infectious Diseases, Robert Koch Institute, Berlin, Germany; 4 Department of Epidemiology and Health Reporting, Robert Koch Institute, Berlin, Germany; 5 Department for Infectious Disease Epidemiology, Robert Koch Institute, Berlin, Germany; University of Hong Kong, Hong Kong

## Abstract

**Background:**

In order to detect levels of pre-existing cross-reactive antibodies in different age groups and to measure age-specific infection rates of the influenza A (H1N1) 2009 pandemic in Germany, we conducted a seroprevalence study based on samples from an ongoing nationwide representative health survey.

**Methodology/Principal Findings:**

We analysed 845 pre-pandemic samples collected between 25 Nov 2008 and 28 Apr 2009 and 757 post-pandemic samples collected between 12 Jan 2010 and 24 Apr 2010. Reactive antibodies against 2009 pandemic influenza A (H1N1) virus (pH1N1) were detected using a haemagglutination inhibition test (antigen A/California/7/2009). Proportions of samples with antibodies at titre ≥40 and geometric mean of the titres (GMT) were calculated and compared among 6 age groups (18–29, 30–39, 40–49, 50–59, 60–69, ≥70 years). The highest proportions of cross-reactive antibodies at titre ≥40 before the pandemic were observed among 18–29 year olds, 12.5% (95% CI 7.3–19.5%). The highest increase in seroprevalence between pre- and post-pandemic was also observed among 18–29 year olds, 29.9% (95% CI 16.7–43.2%). Effects of sampling period (pre- and post-pandemic), age, sex, and prior influenza immunization on titre were investigated with Tobit regression analysis using three birth cohorts (after 1976, between 1957 and 1976, and before 1957). The GMT increased between the pre- and post-pandemic period by a factor of 10.2 (95% CI 5.0–20.7) in the birth cohort born after 1976, 6.3 (95% CI 3.3–11.9) in those born between 1957 and 1976 and 2.4 (95% CI 1.3–4.3) in those born before 1957.

**Conclusions/Significance:**

We demonstrate that infection rates differed among age groups and that the measured pre-pandemic level of cross-reactive antibodies towards pH1N1 did not add information in relation to protection and prediction of the most affected age groups among adults in the pandemic.

## Introduction

The 2009 pandemic influenza A (H1N1) emerged in April 2009 and spread rapidly to countries worldwide [1]–[4]. The antigenic distance from seasonally circulating influenza A (H1N1) viruses raised discussion about the level of pre-existing immunity and immunisation strategies [5]. On 29 Apr 2009 the first laboratory confirmed case in Germany was registered. While initially the majority of cases were in young adults and travel related, the pandemic wave at the population level started in autumn in school-aged children and rapidly spread throughout Germany and peaked in middle of Nov 2009 [6], [7]. The pandemic vaccination campaign in Germany started on 26 Oct 2009. The total number of notified cases until the calendar week 17/2010 was 172 499 and the highest notification rates were reported in the age group of 5–14 years and – as in other countries in Europe – elderly adults above 60 years were less frequently reported [8].

This observation seemed plausible in the context of previously circulating H1N1 strains as a potential cause of pre-existing cross-reactive antibodies against pH1N1 [9]. Part of the population had been exposed to descendants of the 1918 H1N1 pandemic virus circulating until 1957, when it was replaced by H2N2, and after 1977, when H1N1 reappeared in humans again [10], [11]. Thus, it was expected that the risk of infection was lower among older individuals. This hypothesis was supported by results of seroprevalence studies demonstrating that cross-reactive antibodies in the samples collected in the pre-pandemic period were more prevalent among the elderly [9], [12]–[18]. However, there was evidence suggesting that the degree of pre-pandemic serological cross-reactivity varied markedly between populations worldwide [17].

In order to detect levels of pre-existing cross-reactive antibodies in different age groups and to measure age specific infection rates of the influenza A (H1N1) 2009 pandemic in Germany, we conducted a seroprevalence study based on samples from an ongoing representative nationwide interview and examination survey for adults that had started 6 months prior to the first registered case of influenza A (H1N1) 2009 in Germany.

## Materials and Methods

### Study population

The German Health Interview and Examination Survey for Adults (DEGS) [19] is a nationally representative health survey of the adult population in Germany. The DEGS survey is a part of the continuous Health Monitoring and was designed to be representative regarding age, sex and region of residence for the non-institutionalized adult population in Germany. The total sample of 7,500 individuals is being collected between Nov 2008 and Nov 2011 as a stratified two-stage cluster sample. Two professionally trained teams each visit 30 sample points (municipalities) per year, which add up to 180 sample points for the whole study. The sample points are distributed over Germany according to federal state and municipality size in order to reflect the distribution of the German population. The study participants fill in questionnaires, pass physical tests, give blood and urine samples, and have a standardized interview by a physician. In the present study, participants from 46 sample points were included. The study was approved by the Ethics Committee of Charité, University Medicine, Berlin, Germany.

### Sera collection

The pre-pandemic sera were drawn between 25 Nov 2008 and 28 Apr 2009 and post-pandemic sera were drawn between 12 Jan 2010 and 24 April 2010. The sera represent all DEGS study participants of these periods from whom serum samples were available for analysis.

The regions that were covered in this study are distributed across Germany.

### Laboratory procedures

Serum samples were analysed for antibodies to pH1N1 by a validated microtiter hemagglutination-inhibition (HI) test as previously described [20], using the reference strain A/California/7/2009 as antigen. Validation of the HI test was performed by comparative studies. Sera obtained from H1N1 (2009) PCR-positive patients were analysed in a comparative study by the National Reference Centre for Influenza (NIC), Germany, and also by the Paul-Ehrlich-Institute, Langen, Germany. Moreover, samples collected from persons vaccinated with the pandemic H1N1 (2009) vaccine were analysed by the National Institute for Biological Standards and Control (NIBSC), London, UK, the Paul-Ehrlich-Institute and the NIC Germany. Both, the national and international validation of the HI test revealed comparable results [21].

As a first step, each serum was treated with receptor-destroying enzyme to inactivate non-specific inhibitors resulting in a final serum dilution of 1∶10. Sera were then diluted serially twofold into microtiter plates. The virus was adjusted to 4 HA units/25 µl. This concentration was verified by back-titration and 25 µl of the virus suspension was added to each of the 96 wells. After incubation at room temperature for 30 min, freshly prepared 0.5% turkey red blood cells were added. The plates were then mixed by using agitation followed by a further incubation at room temperature for 30 min. Positive and negative controls were included on each plate. An international pH1N1 serum standard received from the NIBSC, London, UK, and sera from vaccinated persons served as controls (titres 40 and 1280). The determination of the HI titre was performed by identification of the reciprocal of the last serum dilution which contained non-agglutinated red blood cells. The titre of the international standard was indicated as 1∶183. Using this standard, a titre of 160 was obtained in different runs. Thus, confirming reproducibility of the international standard HI titre in this study.

All samples were tested twice on different days; there were no samples that differed by more than one dilution step. The minimum detection limit was 1∶10 and samples with titre less than 10 were considered negative and were assigned a value 5 for calculations of the geometric mean. For the subsequent analysis, the geometric mean of the two measurements was used as single observation for each sample.

### Data

For detection of sero-prevalence of reactive antibodies we used pre- and post-pandemic sera. Information about age, sex, and residence in Germany was available for all samples. Vaccination status was assessed using information extracted from the vaccination cards: for those with no vaccination cards, the status was self-reported. For those with vaccination cards, information on vaccine type and date of vaccination was available. For the post-pandemic analysis, we used only the samples with vaccination cards. This was done in order to control pandemic vaccine effect on the level of cross-reactive antibodies by excluding these individuals from further calculations.

### Statistical analysis

In both pre-pandemic and post-pandemic samples, we calculated proportions together with 95% confidence intervals (95% CI) of antibodies at titre ≥10 and ≥40. Furthermore, a gender and age group (18–29, 30–39, 40–49, 50–59, 60–69, ≥70 years) weighted overall mean of these proportions was calculated by utilising population numbers from the German 2008 population (source: German Federal Statistical Office). Similarly, an overall GMT was calculated by exponentiating a population weighted mean of the strata specific log(GMT)s. For both the overall proportion and GMT, CIs were calculated by the percentile method based on 999 draws from two-stage bootstrap cluster sampling, where first the sample points (municipalities) were drawn with replacement, then individuals were drawn with replacement within each sample point [22]. We tested for differences in GMT in two groups with *Wilcoxon* test.

For the subsequent regression analysis, only three birth cohorts (after 1976, between 1957 and 1976, and before 1957 corresponding to age groups 18–32, 33–52 and ≥53, respectively) were used in order to increase the power of the analysis. As the observed titre values range over several orders of magnitude we log-transformed the response for variance stabilization. A special problem of the data is that a standard linear regression model for log(titre) does not apply, because a large proportion of the measurements (87.2% in pre-pandemic and 67.4% in post-pandemic samples) are below the detection limit of 1∶10, and hence are left-censored. Instead, we used a Tobit regression model [23] for the analysis of log(titre) with a value just below log(10) as left censoring limit. The effects of sampling period (pre- and post-pandemic), age, sex, and vaccination on log(titre) could now be investigated. Model selection was performed using a manual stepwise forward selection procedure based on p-values from two-sided likelihood ratio tests. Altogether, a *p*-value below 0.05 was considered to be statistically significant. All statistical analyses were performed using the statistical software STATA version 11 [24] and R version 2.12.0 [25].

## Results

A total number of 845 serum samples for the pre-pandemic period and 757 for the post-pandemic period could be included in analysis. The response was 47% which is above average compared to other large population surveys. For the 845 pre-pandemic samples information on vaccination in 318 (37.6%) samples was based on vaccination cards, in 512 (60.6%) on self reporting, and 15 (1.8%) samples had no information on vaccination. From the 757 post-pandemic samples information on vaccination based on vaccination cards was available for 351 (46.4%) – only these were used for further analysis.

Median age in the pre-pandemic sample was 54 years (range 18–86, mean 51.3, SD ±17.0) and in the post-pandemic it was 47 years (range 18–85, mean 47.5, SD ±16.7). Male to female ratio in the pre-pandemic sample was 0.91 and in post-pandemic 0.86. In the pre-pandemic sample 43.5% (368/845) of individuals had been vaccinated at least once in their lifetime with any seasonal influenza vaccine. In the post-pandemic sample, 30.5% (107/351) of individuals had been vaccinated with a seasonal vaccine and further 4.8% (17/351) had been vaccinated with pandemic vaccine, therefore were excluded from further analysis.

When comparing our study population to the general population in Germany, no significant differences regarding sex were found. However there were significant differences in age distribution (Table S1). This is why weighting procedures have been performed in parts of the analysis.

### Pre-pandemic samples

**Figure 1:**
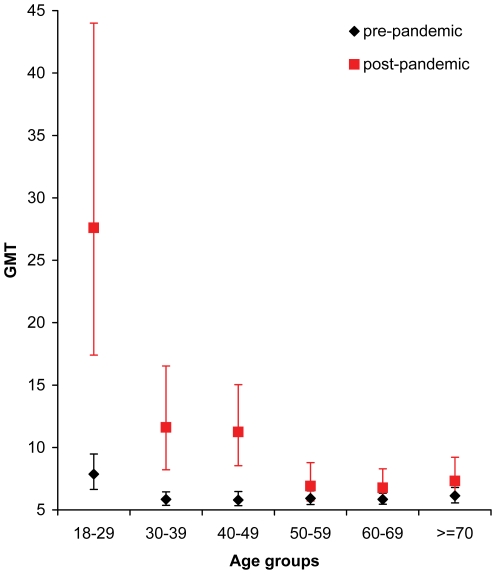
GMT by age group in pre-pandemic and post-pandemic period. In the six age groups in the pre-pandemic sample the GMT ranged from 5.8 to 7.9. GMT in those aged 18 to 29 years (7.9) in comparison to those 30 years of age and older (5.9) was significantly higher. When comparing pre- and post-pandemic results, age groups 18–29, 30–39 and 40–49 had significant increase in GMT with highest increase in the age group 18–29 years.

In the pre-pandemic sample the measured antibody titres ranged from 10 to 640. The weighted overall GMT in pre-pandemic sample was 6.2 (95% CI 5.8–6.7). In the six age groups the GMT ranged from 5.8 to 7.9 as shown in Figure 1 and displayed in Table S2. GMT in those aged 18 to 29 years (7.9) in comparison to those 30 years of age and older (5.9) was significantly higher (*Wilcoxon* test, p <0.001).

The overall proportion of cross-reactive antibodies in the pre-pandemic sample was estimated to be 4.8% (95% CI 2.8–7.0%) at titre ≥40. Those aged 18–29 had the highest proportions of cross-reactive antibodies at titre ≥10 and ≥40 (calculations for titre ≥10 are included in the Table S3). The frequencies and proportions of samples with antibody titre ≥40 in the age groups are displayed in Table 1. In the age group 18–29 years there was no significant difference in proportions of pre-pandemic cross-reactive antibodies at titre ≥40 in samples collected in Nov 2008–Feb 2009 and in samples collected in Mar–Apr 2009 (p = 0.88), the proportion were 12.2% (95% CI 6.3–19.8) and 11.6% (95% CI 3.9–25.1), respectively.

**Table 1:** Number and proportion of observations with reactive antibody titre ≥40 by age groups in pre- and post-pandemic samples and difference in proportions between pre- and post-pandemic samples.

	Pre-pandemic		Post-pandemic		Difference
Age groups (years)	N/Total	% (95%CI)	N/Total	%, (95%CI)	%, (95%CI)
18–29	16/128	12.5 (7.3–19.5)	28/66	42.4 (30.3–55.2)	29.9 (16.7–43.2)
30–39	3/98	3.1 (0.6–8.7)	11/51	21.6 (11.3–35.3)	18.5 (6.7–30.3)
40–49	3/132	2.3 (0.5–6.5)	14/68	20.6 (11.7–32.1)	18.3 (8.4–28.3)
50–59	7/167	4.2 (1.7–8.4)	6/59	10.2 (3.8–20.8)	6.0 (−2.3–14.3)
60–69	7/199	3.5 (1.4–7.1)	2/48	4.2 (0.5–14.3)	0.6 (−5.6–6.9)
≥70	3/121	2.5 (0.5–7.1)	2/42	4.8 (0.6–16.2)	2.3 (−4.7–9.3)

### Post-pandemic samples

The measured antibody titres in the post-pandemic sample ranged from 10 to 1280. The overall GMT in the group was 10.6 (95% CI 8.6–12.8). The age groups 18–29, 30–39, and 40–49 had a significant increase in GMT with the highest increase in the group aged 18–29. GMT by age group in the samples from the post-pandemic period is illustrated in Figure 1 and displayed in Table S2.

Again, those aged 18–29 had highest proportions of antibodies as well as the highest infection rates corresponding to observed differences between pre- and post-pandemic titres (Table 1).

### Pre- and post-pandemic comparison in 3 birth cohorts

**Figure 2:**
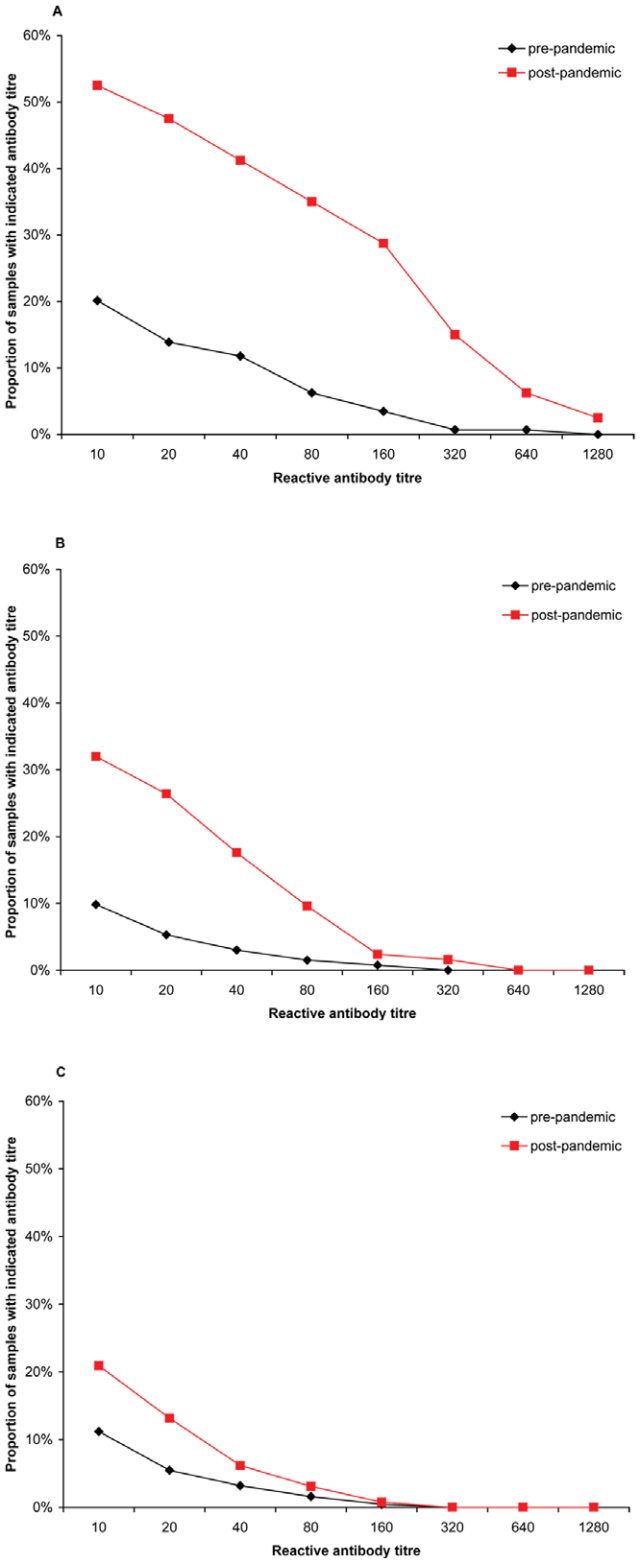
Reverse cumulative distribution curves by birth cohort. Reverse cumulative distribution curves for pre-pandemic and post-pandemic samples by birth cohorts A) after 1976 (18–32 years), B) between 1957 and 1976 (33–52 years), C) before 1957 (≥53 years) and measured antibody titres.

The birth cohort born after 1976 had the highest proportion of antibodies at titre ≥40 in pre- and post-pandemic groups, as well as the highest increase in seroprevalence, 29.4 (95% CI 17.4–41.5%). Calculations for proportions of antibodies at titre ≥40 and GMT by three birth cohorts are displayed in Table S4 and S5, respectively. This result is supported by a reverse cumulative distribution curve shown in Figure 2, which indicates a rapid decline at higher titres in the birth cohort before 1957.

### Tobit regression model

The Tobit regression model contained 1164 observations, of which 953 observations were left-censored. The final model obtained from the model selection procedure contained age, sampling period, and vaccination status as main effects together with an interaction between the age group and the sampling period (the likelihood ratio test for the interaction term was p = 0.005). We estimated that the GMT of the uncensored titre between pre- and post-pandemic period increased by a factor of 10.2 in the birth cohort born after 1976, 6.3 for those born between 1957 and 1976, and as well as 2.4 in those born before 1957. Furthermore, those vaccinated at least once in their lifetime against seasonal influenza had an overall higher titre compared to those not vaccinated, i.e. by factor of 1.9. No interaction between the vaccination and sampling period or age existed, i.e. the increase is the same in pre- and post-pandemic samples. Results of the Tobit regression are summarized in Table 2.

**Table 2:** Investigated effects of age group, sampling period and vaccination on titre using the final Tobit regression model.

Increase in titre:	Factor	95% CI
between pre- and post-pandemic period in age group 18-32	10.2	5.0–20.7
between pre- and post-pandemic period in age group 33–52	6.2	3.3–11.9
between pre- and post-pandemic period in age group ≥53	2.4	1.3–4.3
if vaccinated with seasonal vaccine	1.9	1.3–2.7

The table shows increase in uncensored titre

## Discussion

By analysing samples from a representative nationwide health survey collected in the year preceding the start of the pandemic, we show that the level of pre-existing antibodies at titre ≥40 cross-reacting with the pandemic influenza (H1N1) 2009 virus ranged between 2.3, and 12.5%, depending on age group. The highest proportions of cross-reactive antibodies before the pandemic were observed among 18–29 year olds.

Our findings of higher titres of cross-reactive antibodies among young adults is in contrast to other studies showing higher levels of pre-pandemic cross-reactive antibodies among elderly [9], [12]–[18]. However, published findings vary markedly among different studies. In the US it was found that cross-reactive antibodies were more prevalent in those older than 60 years of age [13], while in Finland only those 80 years and older had high level of pre-existing cross reactive antibodies [9]. As only a few serum samples from individuals older than 80 years (oldest participant 86 years) were included among the pre-pandemic sample in our study, we cannot exclude, that individuals who were born in the years after 1918 have higher pre-existing cross-reactive antibodies. The presence of cross-reactive antibodies in other age groups varies also between different studies. In Italy, UK, and Australia there is some level of pre-existing cross-reactive antibodies found among all age groups with a common trend of higher proportions among older individuals [14], [15], [26], while in Finland, Norway, and US there was only little evidence of cross-reactive antibodies in other age groups than elderly [9], [13], [27]. In Hong Kong only minor levels of pre-pandemic cross-reactive antibodies in the population with no age-specific trend is reported [28].

These differences might be related to the methodological differences in the type and period of sample collection. We analysed samples collected over a 6 month-period directly prior to the start of the pandemic in Germany; in Italy samples from 2003–2004 [15] and in Finland samples from 2004–2005 were analysed [9]. Our analysis stratified by three and six age groups, respectively, suggest that also recently circulating H1N1 strains and vaccination history might have influenced the level of cross-reactive antibodies in German adult population. The last season when seasonal H1N1 influenza viruses dominated in Germany and Europe was 2000/2001 and co-dominated in 2007/2008 [29], [30]. Variation in the epidemiology of circulating subtypes between countries might also explain the different findings in the seroprevalence studies [31]–[33]. This has to be taken into account when comparing serological results.

The sources of sera also differ among the studies. Hancock et al. in the US used 660 stored samples from blood donors and vaccination studies and 417 collected human sera [13]. In the UK, 1403 samples from the patients accessing health care were analysed [14] and in Italy 587 samples were obtained from a seroepidemiological study [15]. In Finland, 1031 stored samples from hospital virology laboratory [9], in Hong Kong sera blood donors, hospital outpatients and community study [28] and in Norway age- and geographically representative residual sera from hospital laboratories were analysed [27]. Using samples collected for other purposes might lead to selection bias with overrepresentation of healthy young adults (e.g. vaccination studies) or persons with particular health problems (e.g. patients accessing health care, hospital laboratory, and vaccination studies). These groups might have different exposure to pH1N1 than general population as well as differences in immunological response. In our study we used a subsample from a population based representative nation wide survey.

Another explanation for the variable results might be related to the differences in laboratory procedures; in our study – as well as the study in Finland, Norway and Australia – HI titres were determined [9], [26], [27], while some of the other studies used microneutralisation assays (MN) [12], [13], [16], [28] or both [14], [34]. Due to the problems of reproducibility of the HI as well as MN methods between laboratories the levels of detected antibody titres may differ among studies if methods are not standardized [35], [36].

Our comparison of reactive antibody prevalence against pH1N1 in pre- and post-pandemic sera indicates that in Germany around one third of those aged 18–29 years and around one fifth of those 30–49 years of age were infected with 2009 pandemic influenza A (H1N1). While those 50 years of age and older had no detectable increase in the proportions of reactive antibodies at titre ≥40. However, analysing individual titres using Tobit modelling (i.e. analysing continuous titre as opposite to dichotomized value) with three birth cohorts adjusting for vaccination, we showed that those born before 1957 had a significant increase in the GMT, but that the increase was the smallest in the three birth cohorts. A similar study from Canada observed the lowest rate of titre ≥40 in those 50–79 years old after the pandemic [34]. A study by Miller et al. found no measurable difference between pre- and post-pandemic period in England among those 45 years and older [14]. A study by Bandaranayake et al. describes higher infection rates in younger individuals and almost no measurable infection rates among elderly [18].

In the literature the presence of cross-reactive antibodies among the elderly as well as lower infection rates during the pandemic are explained by cross-reactive immunity due to previously circulating influenza A (H1N1) strains. The correlation between the HI and clinical protection has been documented for seasonal influenza viruses and HI titre in the range of 30–40 is generally accepted to be associated with a 50% reduction in the risk of influenza infection or disease in a population [37]. In our study, those over 50 years of age had lower proportions of pre-existing cross-reactive antibodies and at the same time lower infection rates. One of the possible reasons for lower risk of infection among older individuals could be pre-existing immunity not detectable by cross-reactive antibodies. This is supported by our results showing that those younger than 50 years of age had highest levels of cross-reactive antibodies prior pandemic as well as highest infection rates. This is in concordance with higher notification rates in those adults younger than 50 in comparison to those over 50 years of age [8]. Other possible explanations are that the older age groups were possibly less affected by pH1N1 infection as they had less contact with younger age groups, or that due to weaker immune response we observe lower reactive antibody levels among elderly. Moreover, infection and vaccination can induce T-cell mediated immune response in humans and it has been shown that some memory T-cell immunity against (H1N1) 2009 is present in the adult population [38]–[40].

Our study has some key characteristics that the aforementioned studies lack. We analysed a representative sample set that was collected 6 months before the pandemic for the pre-pandemic analysis and right after the pandemic for the post-pandemic analysis. Due to the availability of vaccination cards, we were able to control for the effect of pandemic vaccination on measured antibody titres in the post-pandemic period. Moreover, our study is population-based, while other studies used samples from specific groups, e.g. blood donors (healthy donor effect) or hospitalised persons [9], [13]–[15], [27], [28]. We believe that these characteristics are the major strengths of our investigation.

### Limitations

Potential bias introduced by our analysis is that for the pre-pandemic period we used vaccination information based on either recall or vaccination card, but for the post-pandemic period only vaccination cards were used. For those with the vaccination cards the proportion of vaccinated is considerably lower than for those based on recall. We expect this to be due to the influenza vaccination not always being recorded on the vaccination card. Thus for the post-pandemic sample we might underestimate the amount of vaccination. To quantify possible bias we re-fitted the models using only those with vaccination cards for the pre-pandemic sample. We found that the general magnitude and direction of effects was the same as in model with the complete pre-pandemic sample. Significance of all covariates was slightly reduced with vaccination being the only variable not significant anymore. Thus, our strategy provided greater power for the analysis without introducing serious bias. With respect to the regional representativeness: 3 and 7 (of the 16) federal states are not represented in the pre-pandemic and post-pandemic samples, respectively. Still, our results are valid on the German population level, since the smaller federal states are the ones missing and it is fair to assume an equal geographic impact of the pandemic.

As the DEGS study recruited only adults we have no data on the children population. Note also that the DEGS survey is in principle a stratified cluster sample and the clustering was not taken into account in parts of the analysis. Consequently, some of the reported CIs and p-values might be too optimistic, i.e. understating the actual uncertainty. However, since the DEGS study is still ongoing and thus no survey weights are immediately available, the average cluster size is only 25.6 individuals and our subset of post-pandemic vaccination card holders is a greater concern in terms of representativeness than sampling design. We applied additional survey sample methodology for the estimation only of overall proportions and overall GMT.

### Conclusion

We conclude that the infection rates differed among age groups and that the measured pre-pandemic level of cross-reactive antibodies towards pH1N1 did not add information in relation to protection and prediction of the most affected age groups among adults in the pandemic. Further immunological studies and development of better correlates of protection are needed. This would enable more reliable targeting of preventive measures such as vaccination, and would therefore be an important step in preperation for the next pandemic.

## Supporting Information

Table S1 Distribution in age groups and sex in general population and in the study population (n = 1179)
(DOC)

Table S2 GMT by age groups in pre- and post-pandemic samples by age groups
(DOC)

Table S3 Number and proportion of observations with reactive antibody titre ≥10 by age groups in pre- and post-pandemic samples
(DOC)

Table S4 Number and proportion of observations with reactive antibody titre ≥40 by three birth cohorts in pre-pandemic and post-pandemic samples and difference in proportions between pre- and post-pandemic samples
(DOC)

Table S5 GMT by three birth cohorts in pre- and post-pandemic samples
(DOC)
