# Supplementary material for: Prevalence of Antibodies to 2009 Pandemic Influenza A (H1N1) Virus in German Adult Population in Pre- and Post-Pandemic Period
Source: PLoS One. 2011 Jun 20;6(6):e21340. doi: 10.1371/journal.pone.0021340 (PMC3119048; doi:10.1371/journal.pone.0021340)
Supplement: Table S1 — Distribution in age groups and sex in general population and in the study population (n = 1179) (DOC) [file pone.0021340.s001.doc]

Table S1. Distribution in age groups and sex in general population and in the study population (n=1179)

|  | General population | | | Study population | | |
| --- | --- | --- | --- | --- | --- | --- |
| Age groups (years) | % total | % male | % female | % total | % male | % female |
| 18-29 | 17.3% | 18.1% | 16.5% | 16.5% | 16.2% | 16.7% |
| 30-39 | 15.1% | 15.8% | 14.4% | 12.6% | 11.9% | 13.3% |
| 40-49 | 20.4% | 21.5% | 19.4% | 17.0% | 16.2% | 17.7% |
| 50-59 | 16.5% | 17.0% | 16.1% | 19.2% | 17.8% | 20.4% |
| 60-69 | 13.7% | 13.7% | 13.7% | 21.0% | 22.6% | 19.5% |
| ≥70 | 17.0% | 13.9% | 19.8% | 13.8% | 15.4% | 12.4% |
| Total |  | 48.5% | 51.5% |  | 47.2% | 52.8% |
