# Supplementary material for: Prevalence of Antibodies to 2009 Pandemic Influenza A (H1N1) Virus in German Adult Population in Pre- and Post-Pandemic Period
Source: PLoS One. 2011 Jun 20;6(6):e21340. doi: 10.1371/journal.pone.0021340 (PMC3119048; doi:10.1371/journal.pone.0021340)
Supplement: Table S2 — GMT by age groups in pre- and post-pandemic samples by age groups (DOC) [file pone.0021340.s002.doc]

Table S2. GMT by age groups in pre- and post-pandemic samples by age groups

|  | Pre-pandemic | | Post-pandemic | |
| --- | --- | --- | --- | --- |
| Age groups (years) | N | GMT (95% CI) | N | GMT (95% CI) |
| 18-29 | 128 | 7.9 (6.6-9.5) | 66 | 27.6 (17.4-44.0) |
| 30-39 | 98 | 5.8 (5.4-6.5) | 51 | 11.6 (8.21-16.5) |
| 40-49 | 132 | 5.8 (5.3-6.5) | 68 | 11.2 (8.5-15.0) |
| 50-59 | 167 | 5.9 (5.4-6.5) | 59 | 6.9 (5.7-8.8) |
| 60-69 | 199 | 5.8 (5.5-6.3) | 48 | 6.8 (5.7-8.3) |
| >=70 | 121 | 6.1 (5.6-6.8) | 42 | 7.3 (6.1-9.2) |
