# Supplementary material for: Prevalence of Antibodies to 2009 Pandemic Influenza A (H1N1) Virus in German Adult Population in Pre- and Post-Pandemic Period
Source: PLoS One. 2011 Jun 20;6(6):e21340. doi: 10.1371/journal.pone.0021340 (PMC3119048; doi:10.1371/journal.pone.0021340)
Supplement: Table S3 — Number and proportion of observations with reactive antibody titre ≥10 by age groups in pre- and post-pandemic samples (DOC) [file pone.0021340.s003.doc]

Table S3. Number and proportion of observations with reactive antibody titre ≥10 by age groups in pre- and post-pandemic samples

|  | Pre-pandemic | | Post-pandemic | |
| --- | --- | --- | --- | --- |
| Age groups (years) | N/Total | % (95%CI %) | N/Total | % (95%CI %) |
| 18-29 | 28/128 | 21.9 (15.1-30.0) | 35/66 | 53.3 (40.3-65.4) |
| 30-39 | 11/98 | 11.2 (5.7-19.2) | 18/51 | 35.3 (22.4-49.9) |
| 40-49 | 12/132 | 9.1 (4.8-15.3) | 26/68 | 38.2 (26.7-50.8) |
| 50-59 | 15/167 | 9.0 (5.1-14.4) | 9/59 | 15.3 (7.2-27.0) |
| 60-69 | 19/199 | 9.5 (5.8-14.5) | 9/48 | 18.8 (8.9-32.6) |
| ≥70 | 19/121 | 15.7 (9.7-23.4) | 12/42 | 28.6 (15.7-44.6) |
