# Supplementary material for: Prevalence of Antibodies to 2009 Pandemic Influenza A (H1N1) Virus in German Adult Population in Pre- and Post-Pandemic Period
Source: PLoS One. 2011 Jun 20;6(6):e21340. doi: 10.1371/journal.pone.0021340 (PMC3119048; doi:10.1371/journal.pone.0021340)
Supplement: Table S4 — Number and proportion of observations with reactive antibody titre ≥40 by three birth cohorts in pre-pandemic and post-pandemic samples and difference in proportions between pre- and post-pandemic samples (DOC) [file pone.0021340.s004.doc]

Table S4. Number and proportion of observations with reactive antibody titre ≥40 by three birth cohorts in pre-pandemic and post-pandemic samples and difference in proportions between pre- and post-pandemic samples

|  | Pre-pandemic | | Post-pandemic | | Difference |
| --- | --- | --- | --- | --- | --- |
| Age groups (years) | N/Total | % (95% CI) | N/Total | % (95% CI) | % (95% CI) |
| 18-32 | 17/144 | 11.8 (7.0-18.2) | 33/80 | 41.3 (30.4-52.8) | 29.4 (17.4-41.5) |
| 33-52 | 8/264 | 3.0 (1.3-5.9) | 22/125 | 17.6 (11.4-25.4) | 14.6 (7.6-21.6) |
| ≥53 | 14/437 | 3.2 (1.8-5.3) | 8/129 | 6.2 (2.7-11.9) | 3.0 (-1.5-7.5) |
