# Supplementary material for: Prevalence of Antibodies to 2009 Pandemic Influenza A (H1N1) Virus in German Adult Population in Pre- and Post-Pandemic Period
Source: PLoS One. 2011 Jun 20;6(6):e21340. doi: 10.1371/journal.pone.0021340 (PMC3119048; doi:10.1371/journal.pone.0021340)
Supplement: Table S5 — GMT by three birth cohorts in pre- and post-pandemic samples (DOC) [file pone.0021340.s005.doc]

Table S5. GMT by three birth cohorts in pre- and post-pandemic samples

|  | Pre-pandemic | | Post-pandemic | |
| --- | --- | --- | --- | --- |
| Age groups (years) | N | GMT (95% CI) | N | % (95% CI) |
| 18-32 | 144 | 7.6 (6.5-8.9) | 80 | 26.2 (17.3-39.6) |
| 33-52 | 265 | 5.9 (5.5-6.3) | 125 | 9.9 (8.1-12.1) |
| ≥53 | 437 | 5.9 (5.6-6.2) | 129 | 7.0 (6.1-7.9) |
